# A Fiber Alginate Co-culture Platform for the Differentiation of mESC and Modeling of the Neural Tube

**DOI:** 10.3389/fnins.2020.524346

**Published:** 2021-01-12

**Authors:** Orla M. Fannon, Angela Bithell, Benjamin J. Whalley, Evangelos Delivopoulos

**Affiliations:** ^1^School of Biological Sciences, University of Reading, Reading, United Kingdom; ^2^School of Pharmacy, University of Reading, Reading, United Kingdom

**Keywords:** alginic acid, stem cell, germ layers, retinoic acid, tretinoin, embryoid bodies, hydrogels, co-culture techniques

## Abstract

Alginate hydrogels are a commonly used substrate for *in vitro* 3D cell culture. These naturally derived biomaterials are highly tunable, biocompatible, and can be designed to mimic the elastic modulus of the adult brain at 1% w/v solution. Recent studies show that the molecular weight of the alginate can affect cell viability and differentiation. The relationship between the molecular weight, viscosity and ratio of G:M monomers of alginate hydrogels is complex, and the balance between these factors must be carefully considered when deciding on a suitable alginate hydrogel for stem cell research. This study investigates the formation of embryoid bodies (EB) from mouse embryonic stem cells, using low molecular weight (LMW) and high molecular weight (HMW) alginates. The cells are differentiated using a retinoic acid-based protocol, and the resulting aggregates are sectioned and stained for the presence of stem cells and the three germ layers (endoderm, mesoderm, and ectoderm). The results highlight that aggregates within LMW and HMW alginate are true EBs, as demonstrated by positive staining for markers of the three germ layers. Using tubular alginate scaffolds, formed with an adapted gradient maker protocol, we also propose a novel 3D platform for the patterned differentiation of mESCs, based on gradients of retinoic acid produced *in situ* by lateral motor column (LMC) motor neurons. The end product of our platform will be of great interest as it can be further developed into a powerful model of neural tube development.

## Introduction

Traditional cell culture on planar surfaces (e.g., tissue culture plates) does not precisely capture the multifaceted *in vivo* microenvironment. However, complex features of the *in vivo* niche, such as growth factor gradients, different modes of cell migration and cell-matrix interactions, can be more accurately modeled in a 3D biomaterial scaffold. This approach holds the potential to improve our understanding of the temporal and spatial processes involved in creating the diversity of cell types in the human body (Han et al., 2014).

Alginate is a plant-derived biomaterial isolated from the extracellular matrix (ECM) of brown algae (Augst et al., 2006). Alginate hydrogels are cytocompatible, tunable, and degradable biomaterials commonly used as *in vitro* 3D tissue engineering platforms. Previous studies have demonstrated that alginate hydrogels can support the growth of human intestinal organoids (Capeling et al., 2018) and the differentiation of encapsulated embryonic stem cells (ESCs) (Zhang et al., 2013). Alginates have a wide range of compositions and structures depending on the plant species from which the alginic acid is isolated, hence their properties are highly tunable (e.g., viscosity, Young’s modulus) (Matyash et al., 2011; Jeon et al., 2013). The elasticity of alginate hydrogels can also be adjusted to 1.5 kPa, mimicking the elastic modulus of the white and grey matter (Budday et al., 2015). Alginate is well defined but not inherently permissive to cells. However, it may be blended with a variety of ECM-based proteins and polysaccharides, such as gelatin, chitosan, and peptides, in order to allow cell adhesion and cell-matrix interactions (Han et al., 2008; Dalheim et al., 2016; Di Giuseppe et al., 2018).

Alginate hydrogels have been extensively used in the differentiation of stem cells. Maguire et al. (2006) and Wang et al. (2009) encapsulated ESCs in alginate beads to derive functional hepatocytes. Furthermore, human ESCs and human adipose-derived SCs have been differentiated within alginate hydrogels to pancreatic islet-like cells and neural cells, respectively (Khosravizadeh et al., 2014; Richardson et al., 2014). Bozza et al. (2014) demonstrated that alginate beads, blended with fibronectin (Fn) or hyaluronic acid (HA) are an efficient platform for the neural induction of murine ESCs, while Purcell et al. (2009) synthesized an alginate-poly-L-lysine scaffold to similar effect. Neurons of a specific subtype (e.g., dopaminergic, GABAergic) can also be derived, by using alginate hydrogels in co-culture platforms or by including soluble growth factors, such as retinoic acid (RA), in the culture medium (Addae et al., 2012; Kim et al., 2013; Delivopoulos et al., 2015). Frequently, these studies report that alginate-encapsulated ESCs form cell aggregates, have high viability at the end of the culture period and depending on the protocol produce a high proportion of specific cells relative to EB controls. However, no study has yet demonstrated that ESC-derived aggregates within alginate hydrogels are comprised of cells from all three germ layers and are thus canonical EBs.

An abundance of neural differentiation protocols rely on the inclusion of growth factors in the medium. For example, RA and sonic hedgehog (SHH) or SHH agonists are commonly used *in vitro* to emulate the developmental differentiation pathway of motor neurons and astrocytes (Wichterle and Peljto, 2008; Hu and Zhang, 2009; Qu et al., 2014; Juneja et al., 2020). However, it is challenging to engineer complex structures (e.g., organoids) by using techniques that depend largely on the global application of chemical signals. It is more efficient to exploit paracrine effects either in microfluidic platforms (Yang et al., 2015) or in co-culture paradigms, where a mature cell-type regulates the induction of immature cells, usually ESCs (Chou and Modo, 2016; Farrukh et al., 2018). This form of localized signaling is found in the development of the neural tube, when medial limb-innervating lateral motor column neurons (LMCm-MNs) synthesize retinoic acid, *via* Raldh2, that in turn determines the cell fate of later developing, lateral LMC (LMCl) MNs and guides their axonal growth (Sockanathan and Jessell, 1998; Ji et al., 2006).

This study describes the guided differentiation of mouse ESC-derived aggregates, encapsulated in alginate scaffolds. We demonstrate that alginate hydrogels of different molecular weights (high and low) are capable of supporting mESC differentiation into EB-like aggregates, containing cells that express markers from the three germ layers, using a retinoic acid (RA) based differentiation protocol (Montgomery et al., 2015). This validates the use of alginates in a variety of studies, ranging from germ layer organization to ESC neural induction. As a proof of concept, we engineered a fiber co-culture, alginate platform with mESCs and LMCm-MNs acting as an *in situ* source of RA. Inside the tubular scaffolds, the LMCm motor neurons regulated the neural induction of pluripotent mESC. The proposed co-culture platform is not yet a fully functional 3D spinal cord model however, we believe it has significant potential to become a versatile *in vitro* tool in studying patterned differentiation and neural tube development.

## Materials and Methods

### Maintenance of Mouse Embryonic Stem Cells and Fibroblasts

Mouse embryonic fibroblasts (MEFs) and ESCs were cultured on gelatin-coated tissue culture flasks (0.1%). CGR8 mouse ESCs from passage 10–20 were used for alginate characterization and co-culture experiments and were cultured in CGR8 medium composed of Dulbecco’s modified eagle medium (DMEM)-high glucose, 10% ES-qualified fetal bovine serum (FBS), 100 units/ml penicillin, 100 μg/ml streptomycin, 2 mM L-glutamine, 100 μM beta-mercaptoethanol (βME), and 1,000 units of LIF/ml. Medium was changed daily to maintain pluripotency and ESCs were passaged at a ratio of 1:8 at 80% confluence (approximately every 2 days).

HGF11 ESCs were cultured on MEF feeder layers (see below) in HGF11 medium: DMEM-high glucose, 10% FBS, 100 units/ml penicillin, 100 μg/ml streptomycin, 2 mM L-glutamine, 1X non-essential amino acids (NEAA), 100μM β-ME, and 1,000 units of LIF/ml. Medium changes and passaging were performed as CGR8 ESCs above.

MEFs (strain CF1) were cultured as feeder layers for the HGF11 ESC line in MEF medium: DMEM-high glucose, 10% FBS, 100 units/ml penicillin, 100 μg/ml streptomycin, and 2 mM L-glutamine. Cells were passaged at a ratio of 1:2 at 80% confluence. At passage 6, confluent cells were inactivated with 10 μg/mL Mitomycin-C treatment for 2 h, washed three times with Dulbecco’s Phosphate Buffered Saline (DPBS) and cultured for up to 10 days. MEF medium was replaced with ESC medium 30 min before seeding with HGF11 ESCs (Fannon, 2018).

### Separation of HGF11 ESCs From Feeder Layers

The presence of MEFs in EB suspension cultures reduces differentiation efficiency. To prevent carryover to differentiation experiments, HGF11 ESCs were trypsinised and replated onto gelatin-coated 100 mm dishes (0.1%) in ADFNK medium (Advanced DMEM-F12:NBM (1:1) with 10% knockout serum replacement, 100 units/mL penicillin, 100 μg/mL streptomycin, 2 mM L-glutamine, and 100 μM βME) for 30–45 min where remaining MEFs adhered and ESCs in suspension were aspirated and used for downstream experiments.

### ESC Neuralization

ESCs differentiated using the EB suspension method served as a control condition for alginate hydrogel experiments. ESCs were suspended at 5 × 10^4^ cells/ml in ADFNK in 100 mm suspension dishes and cultured for 6 days with 1 μM RA added on days 2 and 4 (in specific preparations 1.5 μM purmorphamine was also added, to obtain a positive control for motor neuron differentiation). To change the medium, EBs were centrifuged to pellet (3 min, 200 rcf) and resuspended in 10 ml ADFNK supplemented with 1 μM RA.

### EB Dissociation and Monolayer Culture for Immunostaining

On day 6 of differentiation, EBs were washed in DPBS, re-pelleted (as section “ESC Neuralization”) and resuspended in 5 ml of 0.25% trypsin-EDTA (1X) on a rocker for 5–10 min at room temperature. 5 ml of ADFNK was added to dilute the trypsin-EDTA before centrifugation (5 min, 200 rcf). Cells were resuspended in 5 ml of ADFNK, passed through a 70 μm cell strainer, counted using trypan blue exclusion, and replated onto laminin-coated (2 μg/cm^2^) coverslips for 2 days in ADFNK medium with 5 ng/ml glial cell line-derived neurotrophic factor (GDNF). Cells were fixed for 20 min in 3.7% paraformaldehyde (PFA), washed once in DPBS, and processed for immunocytochemistry (ICC).

### Differentiation of Motor Neurons From HGF11 ESCs

HGF11 ESCs were differentiated using a published differentiation protocol from Wichterle and Peljto (2008). Briefly, HGF11 ESCs were differentiated toward a MN lineage by EB suspension culture, adding 1 μM RA and 1.5 μM purmorphamine on days 2 and 4. This protocol generates 30–50% MN populations by day 6. On day 6, EBs were collected, dissociated by trypsinization and replated onto laminin-coated coverslips at 1 × 10^5^ cells/cm^2^ for ICC, or 17.5 × 10^5^ cells/cm^2^ for ELISA. For ICC, cells were cultured for 2 days in ADFNK supplemented with 10 ng/ml GDNF, then fixed and used for immunostaining. Control conditions were: HGF11 ESCs differentiated with 1 μM RA only or CGR8 wildtype ESCs differentiated to MNs as described (Delivopoulos et al., 2015).

### Quantification of Endogenous Retinoid Concentrations

HGF11 ESCs were differentiated as above and replated at 1 × 10^5^ cells/cm^2^ onto laminin-coated coverslips. The cells were cultured for 6 days after replating in ADFNK supplemented with 5 ng/ml GDNF to enhance the maturation and survival of MNs (Wichterle and Peljto, 2008). Half of the medium was changed every 2 days, allowing 48 h before samples were collected for competitive ELISA for RA and Raldh2 (MyBioSource MBS7237205, MBS 706971). Data were tested for outliers using the ROUT method at 10%, tested for normality using the Shapiro–Wilk method, and analyzed using a one-way ANOVA and multiple comparisons were conducted using Tukey’s *post hoc* analysis.

### Cryosectioning (EB, Alginate Beads, and Alginate Fiber Co-cultures)

Alginate beads, fiber co-cultures and EBs were fixed at day 6 of differentiation for 1 h in 3.7% PFA, cryoprotected for 4–6 h in 30% sucrose solution, embedded in optimum cutting temperature compound (OCT) and stored at −80°C. Samples were sectioned at 15–30 μm, collected on gelatin-coated microscope slides, dried overnight at 37°C and stored at 4°C. Slides were coated by washing in a 0.2% gelatin: 0.02% chromium potassium sulfate dodecahydrate solution; slides were dipped into the solution five times for 5 s, then air-dried for 1 min; this was repeated five times. Slides were dried overnight at room temperature.

### Immunocytochemistry

Samples were blocked and permeabilized in 20% normal goat serum (NGS) in 0.05% Triton-X-100 in DPBS for 1.5 h at room temperature. Primary antibodies were applied for 1.5 h at room temperature or overnight at 4°C and secondary antibodies were applied for 2 h at room temperature. Nuclei were counterstained with Hoechst 33342 (1:25,000) for 10 min at room temperature. All washes were in DPBS. For full list of antibodies and concentrations see Supplementary Table 1. Samples were mounted using Vectashield mounting medium and imaged using a Zeiss Axio Imager A1 fluorescence microscope and Axiovision software (v4.0). Negative control tissues used for immunofluorescence analysis were as follows: (a) negative controls for alginate beads: CGR8 were encapsulated in alginate beads and cultured for 6 days in ESC medium with LIF to prevent differentiation, (b) HGF11-derived MN characterization: HGF11 ESCs were cultured on gelatin-coated coverslips in ESC medium until confluent, and (c) HGF11-derived MN GFP/FoxP1 validation: HGF11 ESCs were differentiated using 1 μM RA only. CGR8 ESCs were differentiated with 1 μM RA and 1.5 μM purmorphamine toward a MN lineage.

### Formation of Cell-Encapsulating Alginate Hydrogels

Cell-encapsulating alginate beads were formed by modifying an existing protocol (Bozza et al., 2014). CGR8 were resuspended in 1% w/v alginate: 0.1% gelatin at a cell density of 3 × 10^6^ cells/ml alginate. Two molecular weights of alginate were tested: low molecular weight, low viscosity alginate with a high G:M ratio (hereafter referred to as LMW) and high molecular weight, high viscosity alginate with a low G:M ratio (hereafter referred to as HMW, see Supplementary Table 2 for details). Alginate beads were formed by manually extruding the alginate-cell suspension from a syringe with a 21 g needle into a bath of 100 mM CaCl_2_/10 mM 4-(2-hydroxyethyl)-1-piperazineethanesulfonic acid (HEPES). The alginate beads were polymerized for 5 min at room temperature, washed once with ADFNK, and cultured for 6 days in ADFNK with 1 μM RA added on days 2 and 4. Cell density per bead was calculated by dividing the volume of alginate by the number of beads: LMW beads ∼6 × 10^4^ cells/bead; HMW beads ∼5 × 10^4^ cells/bead. On day 6, beads and EBs (control) were collected and prepared for cryosectioning [see section “Cryosectioning (EB, Alginate Beads, and Alginate Fiber Co-cultures)”].

### Boyden Chamber Co-culture

HGF11 ESCs differentiated to day 6 (see section “Differentiation of Motor Neurons From HGF11 ESCs”) were replated onto laminin-coated coverslips in 12-well plates and alginate beads were separately prepared (day 0 differentiation, see section “Formation of Cell-Encapsulating Alginate Hydrogels”) and transferred into Boyden chambers (8–10 beads per chamber). After 48 h, half of the medium on the HGF11-derived MNs was replaced and each Boyden chamber containing alginate beads was transferred into a well containing HGF11-derived MNs. After 2 days (day 4 alginate differentiation), half of the medium was changed, and on day 6 of alginate differentiation the beads were fixed, cryopreserved and immunostained for markers of the three germ layers (see section “Immunocytochemistry” and Supplementary Table 1).

### Alginate Fiber Co-culture

**FIGURE 1:**
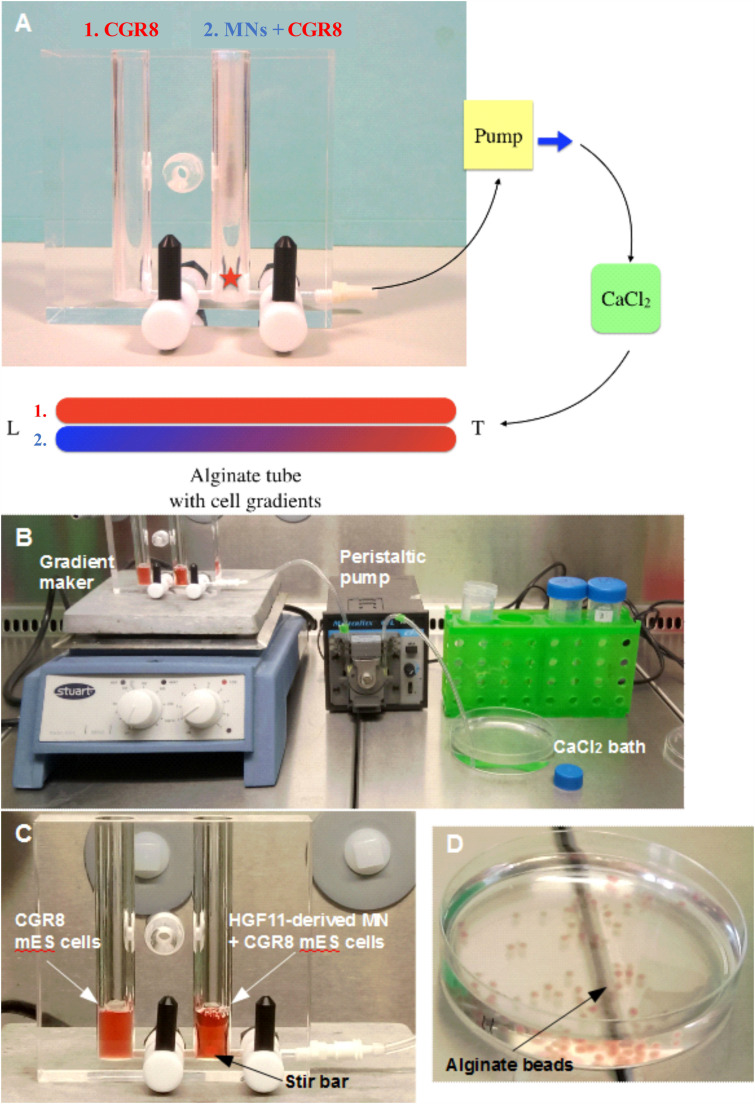
Formation of alginate beads and fibers (tubes). **(A)** The gradient is formed from two alginate: cell solutions: 1. (in red) alginate containing CGR8 ESCs only, and 2. (in blue/red) alginate containing CGR8 ESCs and HGF11 ESC-derived MNs. An equal volume of alginate solutions 1 and 2 are added to chambers 1 and 2, respectively (MN: HGF11-derived MNs, CGR8: wildtype ESCs, Red star: location of stir bar). **(B)** The gradient maker setup and equipment for making the alginate fibers. **(C)** A close-up image of the gradient maker showing the two chambers containing alginate-cell suspension solutions for forming alginate fibers. **(D)** Beads formed from 1 mL of alginate: cell (CGR8 ESCs) suspension by manual extrusion through a 21G needle.

Mature HGF11-derived MNs were co-cultured with CGR8 ESCs inside alginate fibers. Fibers were created with a standard gradient maker (Figure 1A), using the setup shown in Figures 1B,C. Two alginate cell suspensions (1: CGR8 ESCs, 2: HGF11 ESC-derived MNs + CGR8 ESCs) were loaded into the chambers and drawn into a CaCl_2_ bath, *via* the action of the peristaltic pump (Figure 1B) (Fannon, 2018). When the pump was switched on, a volume of alginate from chamber “B” was drawn into the tubing to form the Leading Edge (L). This simultaneously draws an equal volume of cell suspension from chamber 1 into chamber 2, which is then mixed with the cell suspension in chamber 2 (Figure 1C). In this manner, the cell suspension from chamber 1 is continuously drawn into and mixed with the suspension in chamber 2. Whilst the fiber is formed through the action of the peristaltic pump, the cell suspension in chamber 2 is diluted with that drawn from chamber 1, such that the Trailing Edge (T) contains a low concentration of cell suspension 2 and a high concentration of cell suspension 1.

### Quantitative Polymerase Chain Reaction

Alginate beads (section “Formation of Cell-Encapsulating Alginate Hydrogels”) were cultured for 6 days and harvested using 50 mM EDTA, 120 mM sodium chloride (NaCl), and 10 mM Hepes (pH 7.4) for 5 min at room temperature. The aggregate suspension (or EB controls) were centrifuged (3 min, 200 rcf) and RNA was extracted using an Qiagen RNeasy kit, according to the manufacturer’s instructions (Qiagen). RNA concentration was measured using a Nanodrop 2000 spectrophotometer. RNA samples were stored at −80°C. Where necessary, RNA samples were further concentrated using the Qiagen RNeasy MinElute Cleanup kit according to the manufacturer’s instructions. The Qiagen Quantinova Reverse Transcription kit was used to synthesize cDNA and eliminate genomic DNA according to the manufacturer’s instructions (500 ng of template RNA in 2 μl of gDNA removal mix). For negative control reactions, reverse transcriptase was omitted. Primers were designed for Oct-4 (Pou5f1), Nestin (Nes), Pax6 and β-actin (Actb) using Primer3 (Supplementary Table 3) and their efficiency validated using a standard curve and the StepOne software (Applied Biosystems). The positive control was cDNA combined from EBs and cells harvested from alginate beads (4 EB, 4 HMW, and 3 LMW samples). Undifferentiated ESC cDNA was used as an additional negative control. Quantitative polymerase chain reaction (qPCR) was performed using the Qiagen Quantinova SYBR green kit, according to the manufacturer instructions, an Applied Biosystems StepOnePlus Real-Time PCR system and StepOne Software (v2.3). Thermal cycler conditions: 2 min at 95°C, followed by 40 cycles of 5 s at 95°C and 10 s at 60°C.

## Results and Discussion

### ESC-Derived Aggregates Form in Alginate Beads

**FIGURE 2:**
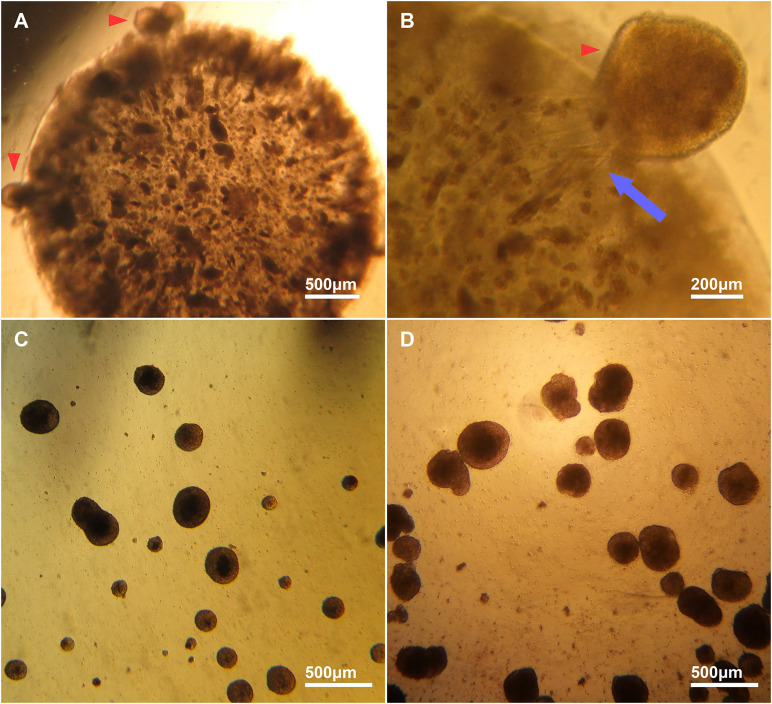
Brightfield images of encapsulated and escaped cell aggregates from alginate and EBs. **(A)** Representative image of aggregate formation by encapsulated ESCs inside an alginate bead. **(B)** A high magnification image of an aggregate migrating out of an alginate bead (red arrowhead). The escaped aggregates migrated out of the beads as whole aggregates, and appear to interact with the alginate matrix, forming striations in the bead (blue arrow). **(C)** Cell aggregates formed inside an alginate bead that have migrated out and are free-floating in medium. **(D)** Canonical EBs are similar in size and shape to cell aggregates that escape from alginate: DMEM beads.

Mouse CGR8 ESCs were encapsulated in alginate beads synthesized in either distilled sterile water (H_2_O) or DMEM (Figure 1D). Cells in the alginate: H_2_O beads did not form aggregates and remained as single cells. Contrastingly, brightfield microscopy analysis of alginate: DMEM beads at day 6 of differentiation revealed the formation of a large number of aggregates by the encapsulated cells (Figure 2A). Cell aggregates interacted with the alginate matrix and many were visible at the edges of the beads (Figure 2A red arrows). Some aggregates were observed migrating out of the alginate beads (Figure 2B red arrow) and floating in the cell culture medium, possibly generating striation marks in the direction of migration (Figure 2B blue arrow). These aggregates (Figure 2C) formed entirely in the bead interior before escaping into the medium and were found to be similar in size and shape to canonical EBs, formed by the cell suspension method (Figure 2D).

Alginate hydrogels polymerize *via* divalent ions, which form ionic bridges between G monomers. In cell culture, monovalent ions in the medium compete with the binding sites on G monomers, causing alginate hydrogels to lose their mechanical stability over time (Drury and Mooney, 2003). This slow de-crosslinking of the alginate hydrogel near its interface with the medium may have contributed to the release of aggregates that were close to the edges of the beads in our experiments. Controlled hydrogel degradation can be an advantage in *in vivo* applications (Wang et al., 2003) however, a change of the mechanical properties of the hydrogel *in vitro* can also affect the differentiation of encapsulated stem cells (Khetan et al., 2013) and therefore should be mitigated by inclusion of CaCl_2_ in the medium when possible.

### Gelatin Concentration Affects Aggregate Shape Whilst Alginate Molecular Weight Does Not Affect Aggregate Formation or Viability

**FIGURE 3:**
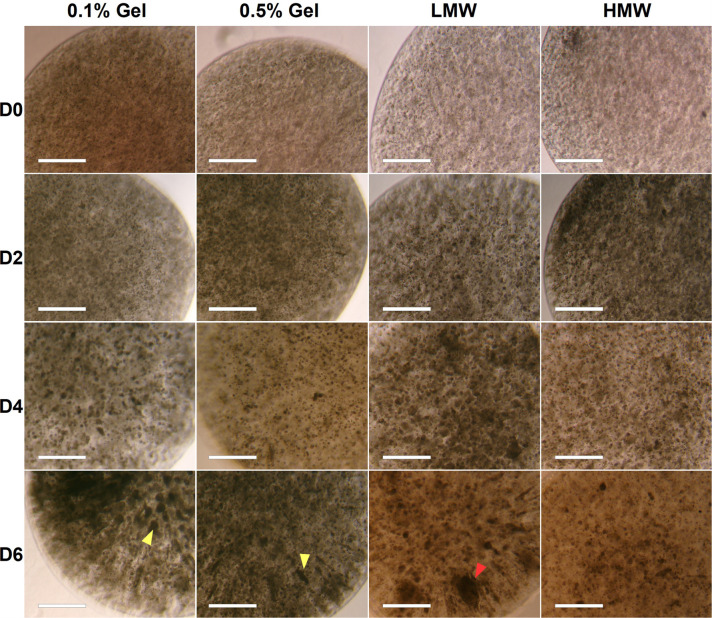
Brightfield images of aggregate formation by encapsulated ESCs in high and low molecular weight alginate (HMW and LMW, respectively) mixed with 0.1 and 0.5% gelatin. Although some aggregates in LMW were very large (∼300 μm, red arrowhead), these were infrequent and the majority of beads contained smaller circular and tubular aggregates (∼100 μm). Scale Bar: 500 μm.

Alginate beads (HMW) containing 0.1 or 0.5% gelatin showed aggregate formation across 6 days of differentiation (left hand panels, Figure 3). The 0.1% gelatin conditions generated spherical aggregates, compared to the elongated aggregates in 0.5% gelatin (Figure 3 yellow arrowheads in D6 bottom left 2 panels). This difference may be due to reduced porosity and permeability of the 0.5% gelatin alginate, preventing matrix displacement by the cells (to form spherical aggregates), and a reduction of nutrient supply and growth factor signaling to the encapsulated stem cells (Lai et al., 2013). Alternatively, local fissures in the material (at points of low G monomer and crosslink concentration) can create pathways for cell escape thus, appearance of ovoid aggregates may be due to cell expansion along the axis of weak material (Wilson et al., 2014). Given that canonical EBs are spherical in shape, the 0.5% gelatin conditions were discontinued, in favor of 0.1% gelatin conditions which supported spherical aggregates.

The effect of alginate molecular weight on aggregate formation was also assessed (see section “Formation of Cell-Encapsulating Alginate Hydrogels”). Aggregates formed within both LMW and HMW scaffolds (0.1% gelatin), by day 4 of differentiation (Figure 3, right hand panels). We expected observable differences in cell aggregation between scaffolds of different molecular weights, due to the higher G-residue content of the LMW alginate (60%) compared to HMW (40%). Since crosslinks form between G-monomers, LMW beads should have a higher elastic modulus, a more stable internal structure and a lower porosity, which would have inhibited aggregate formation and size, due to reduced cell-matrix interactions (Kuo and Ma, 2001). However, both HMW and LMW alginates contained viable aggregates of circular and elongated shape. Furthermore, we observed similar swelling in both types of alginate beads, during the 6 day incubation period. We documented a statistically significant increase in bead diameter for both HMW {one-way ANOVA [*F*(3,8) = 10.9476, *p* = 0.0033]} and LMW {one-way ANOVA [*F*(3,8) = 8.9472, *p* = 0.0062]} alginate, suggesting a gradual water uptake and decrosslinking in all gels (Supplementary Figure 1).

**FIGURE 4:**
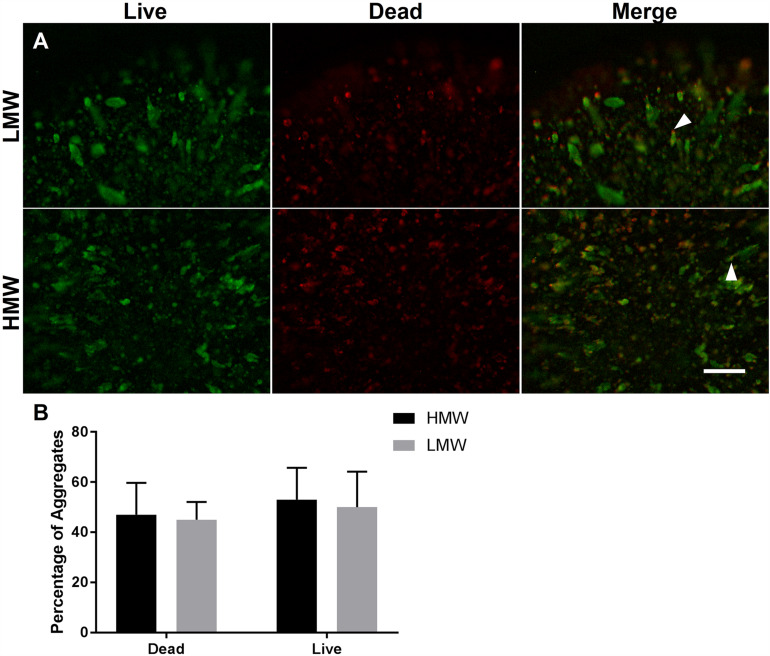
Cell viability of encapsulated ESCs in **(A)** LMW and HMW beads. Alginate beads of LMW and HMW contained both live cells (green) and dead cells (red) on day 6 of differentiation. The merged images demonstrate that aggregates contained a mixture of live and dead cells, with the dead cells localized to the tips of tube/lens shaped aggregates (white arrowheads). **(B)** Quantification of cell viability in LMW and HMW beads. A two-tailed *t*-test revealed no significant difference between the two alginates (*p* = 0.1257). Four technical replicates collected from 2 independent experiments and presented as mean ± SEM. Scale Bar **(A)**: 500 μm.

A live/dead assay revealed live cells in both types of alginate hydrogels by day 6 of differentiation (Figures 4A,B). Live cells were predominantly observed within larger aggregates, whereas dead cells were observed either as single cells or within smaller aggregates. Some elongated aggregates contained dead cells at the tips (Figure 4, white arrowheads), consistent with observations by Wilson et al. (Wilson et al., 2014). This could be due to the localized absence of cell-interactions at the aggregate tips. Quantification of cell viability in LMW and HMW beads revealed no significant difference between the LMW and HMW (two-tailed *t*-test, *p* = 0.1257, *n* = 4 replicates from two independent experiments, Figure 4B). This result is in agreement with a similar study on mesenchymal stem cell differentiation in alginate bioinks of variable molecular weight, where no difference in cell viability was reported between groups (Freeman and Kelly, 2017). The similar viability may be the result of cell-cell interactions that are preserved between the two alginate subtypes, despite the different G:M ratio. Furthermore, EBs are known to have their own elastic modulus which may override the elastic modulus and hydrogel structure differences between the two examined scaffolds, allowing similar viability and aggregate morphology to occur.

### EB-Like Aggregates in Alginate Beads Express Markers of the Three Germ Layers

**FIGURE 5:**
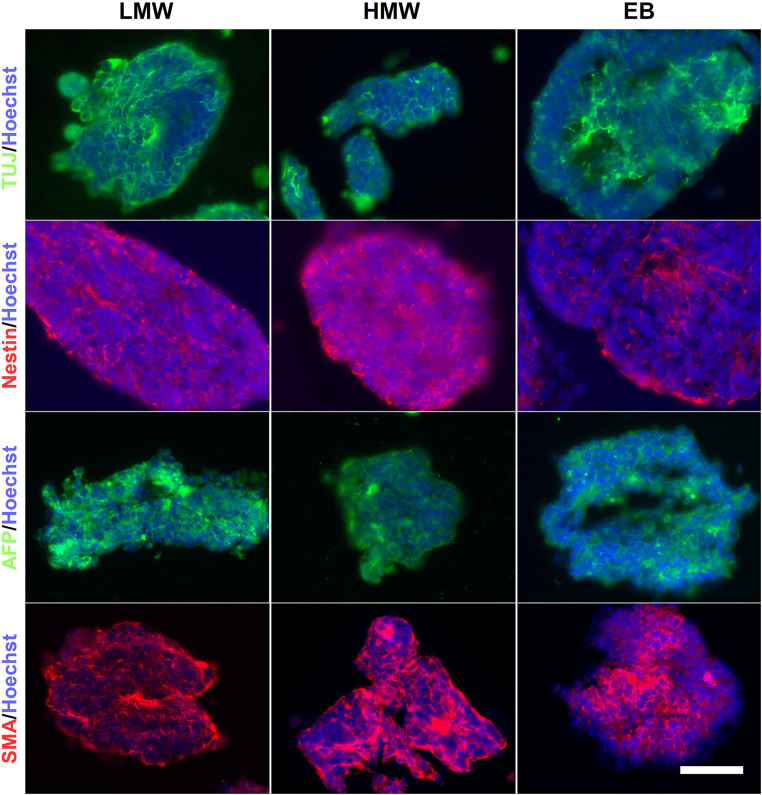
EBs and alginate beads contain cells from the three germ layers. Encapsulated aggregates in LMW and HMW alginate beads and EBs are positive for βIII-tubulin (TUJ1), Nestin, AFP, and SMA indicating the presence of the three germ layers. Scale Bar: 50 μm.

The presence of cells from the three germ layers in alginate beads and compared to EBs was investigated using fluorescence ICC. The results demonstrated that LMW and HMW alginate beads and EB controls contained cells positive for markers of endoderm (AFP), mesoderm (SMA), and ectoderm (nestin, ßIII-tubulin) (Figure 5), providing evidence that both alginate formats support generation of all three germ layers. Alginate beads are shown as suitable scaffolds for neural differentiation, as determined by the presence of neuroectoderm markers Nestin and ßIII-tubulin. Our results are consistent with previous studies using RA to induce neural differentiation in alginate hydrogels (Wang et al., 2009; Li et al., 2011; Bozza et al., 2014) and provide evidence that mouse ESCs encapsulated in LMW and HMW alginate beads form EB-like aggregates, comparable to EBs formed by cell suspension protocols.

**FIGURE 6:**
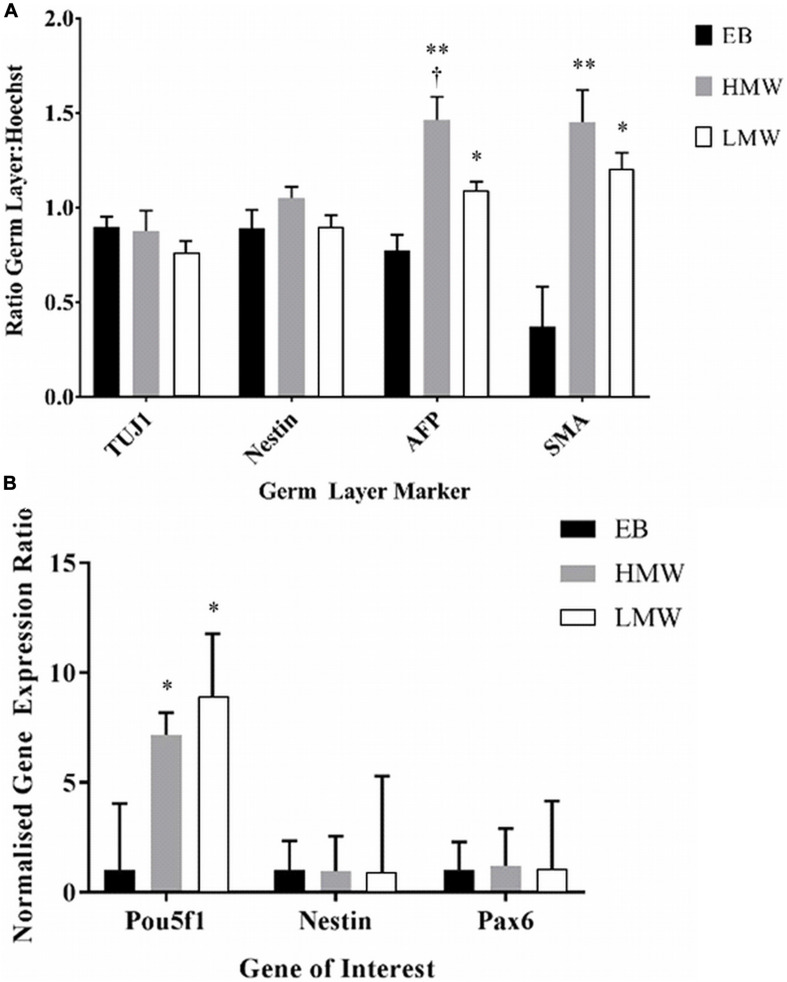
**(A)** Data from pixel quantification of germ layer markers relative to nuclear stain for each differentiation condition (EB, HMW bead, and LMW bead). **(B)** Gene expression for neural and pluripotency markers in alginate beads and EBs. (*p < 0.05, **p < 0.01 versus EBs, *n* = 3). Data from three experiments (*n* = 3 samples in each) and presented as mean and SEM. † indicates statistical significance (*p* < 0.05) versus LMW beads.

Due to the decrosslinking that occurs in alginate beads in cell culture medium, potentially creating a softer elastic modulus at the exterior compared with the interior of the scaffolds, alginate beads and EBs were serially sectioned and immunostained to investigate spatial variations in lineage differentiation across sequential sections of the samples. Immunofluorescence images reveal aggregates positive for all three germ layers, across all sections of LMW and HMW scaffolds, as well as EBs (Supplementary Figures 2, 3). However, the number of aggregates containing cells positive for each germ layer marker may vary across different planes of the samples. For EBs and aggregates encapsulated in HMW beads there appeared to be higher numbers of aggregates that contained TuJ1^+^ cells (ßIII-tubulin antibody) and AFP^+^ cells. Contrastingly, LMW beads appeared to contain higher numbers of encapsulated aggregates that contained Nestin^+^ cells and SMA^+^ cells. To investigate whether the observed variations in germ layer marker presence was significant, pixel counting was used to quantify the area of fluorescence for each marker in all images. To control for the different number of cells in alginate beads relative to EBs, the pixel values for each image were converted to a ratio by dividing the area of the fluorescent signal for each germ layer marker (AFP, SMA, Nestin, and TuJ1) by the area of fluorescent signal for the cell nuclei marker (Hoechst). Figure 6A shows the ratio values of germ layer marker to nuclear staining, for each differentiation condition. For each germ layer marker, the data was analyzed by two-way ANOVA to compare the differentiation condition (EBs, HMW beads, LMW beads) with germ layer presence. The ratio values and analysis indicate a statistically significant higher proportion of AFP^+^ and SMA^+^ cells in HMW and LMW beads relative to EBs (Figure 6A). We would expect an even distribution of germ layer markers across aggregates, as well as symmetry breaking, due to the gradual establishment of the three body axes (AP: anteroposterior, DV: dorsoventral, and LR: left-right) (Fuchs et al., 2012; Turner et al., 2017). Future work will fully elucidate any spatial variability in differentiation and further quantitate the precise composition of ESC aggregates within the alginate.

The expression of neural (Nestin, Pax6) and pluripotency (Pou5f1) markers was also assessed by quantitative PCR to compare the capacity of LMW or HMW alginate beads and EBs in specification of mouse ESCs to a neural fate (Figure 6B). A two-way ANOVA showed no interaction between the differentiation conditions (LMW, HMW, and EBs) and the expression of Pou5f1, Nestin or Pax6 [*F*(4,21) = 1.4, *p* = 0.260, df = 4]. Tukey’s *post hoc* analysis showed that there was a significantly higher expression of Pou5f1 in HMW beads (*p* = 0.047) and in LMW beads (*p* = 0.047) compared to EBs. This result is in agreement with previously reported findings where alginate beads maintained stem cell pluripotency over long periods of time compared to EBs (Siti-Ismail et al., 2008). Despite the increased number of pluripotent stem cells, alginate is still viable as a platform for neural differentiation, since expression levels of neural markers were comparable to EBs.

### LMC Motor Neurons Can Provide a Source of Endogenous Retinoic Acid

When hydrogel scaffolds are used in tissue engineering, morphogens are often applied globally by inclusion in the medium (Gu et al., 2016; Lin et al., 2018). However, it is more efficient to localize the morphogen source close to the differentiating stem cells and exploit paracrine signaling. For example, in neural induction of ESCs a cell organizer can produce stable concentrations of RA, irrespective of medium changes, while addressing the limitation of light-degradation of RA. In the neural tube, LMCm-MNs synthesize retinoic acid to induct later developing MNs. The derivation of LMC-MNs from mouse ESCs was recently achieved by Adams et al. (2015) through the manipulation of the transcription factor Foxp1. In order to have a localized source of cell-derived RA in our system and compare effects with exogenous RA, we used the HGF11 ESC line, derived from transgenic HB9:Foxp1 mice where Foxp1 and GFP expression are under control of HB9. When differentiated toward a MN fate, the expression of Foxp1 in post-mitotic HB9^+^ MNs forces an LMC-MN fate responsible for secretion of RA *in vivo* (Adams et al., 2015).

**FIGURE 7:**
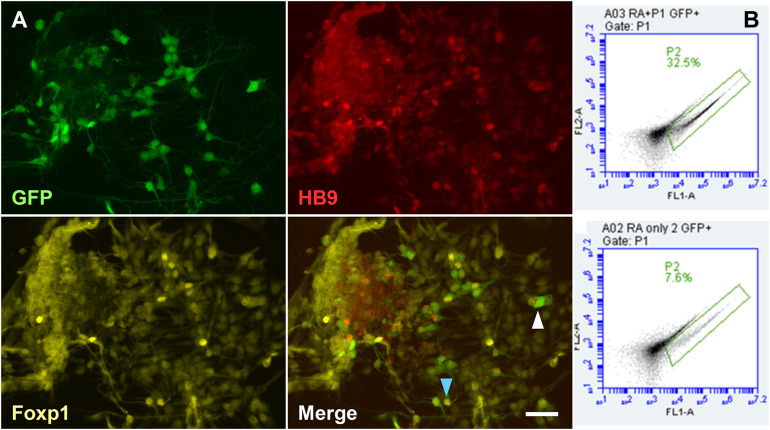
Derivation of LMC-MN from the HGF11 transgenic cell line. **(A)** HGF11-derived MNs were GFP^+^, HB9^+^, and Foxp1^+^. GFP^+^ cells were usually Foxp1^+^ and HB9^+^ (blue arrowhead), with a few exceptions (white arrowhead). *n* = 3 independent experiments. Scale Bar: 20 μm. **(B)** Quantification of GFP^+^ cells *via* flow cytometry. HGF11 ESCs differentiated with RA and purmorphamine (top panel) or just RA (bottom panel). *n* = 3 independent experiments, 10,000 events per condition.

To first confirm LMC-MN differentiation, HGF11-derived MNs were immunostained to demonstrate co-localization of GFP, HB9, and Foxp1 (Figure 7A, blue arrowhead). CGR8 ESC-derived MNs (which generate a medial motor column fate) were used as a negative control and did not contain any GFP^+^ cells (Supplementary Figure 4). Small numbers of GFP^–^:HB9^+^:Foxp1^+^ cells observed in the CGR8 derived MNs is in line with reported low spontaneous differentiation to an LMC fate (Peljto et al., 2010; Adams et al., 2015). The percentage of HGF11-derived GFP^+^ LMC-MNs was assessed by flow cytometry. Approximately 32.5% of cells were GFP^+^ (Figure 7B), consistent with previous reports of 30% by Wichterle et al. (Wichterle and Peljto, 2008) and 40% by Adams et al. (Adams et al., 2015). As an additional control we differentiated HGF11 ESCs without purmorphamine, which is the ventralizing morphogen required to drive a MN fate, and thus we expect lower numbers of GFP^+^ cells from spontaneous generation of low numbers of HB9^+^ MNs. Here we observed 7.6% GFP^+^ cells (Figure 7B bottom panels), consistent with the expected levels (Peljto et al., 2010; Adams et al., 2015).

**FIGURE 8:**
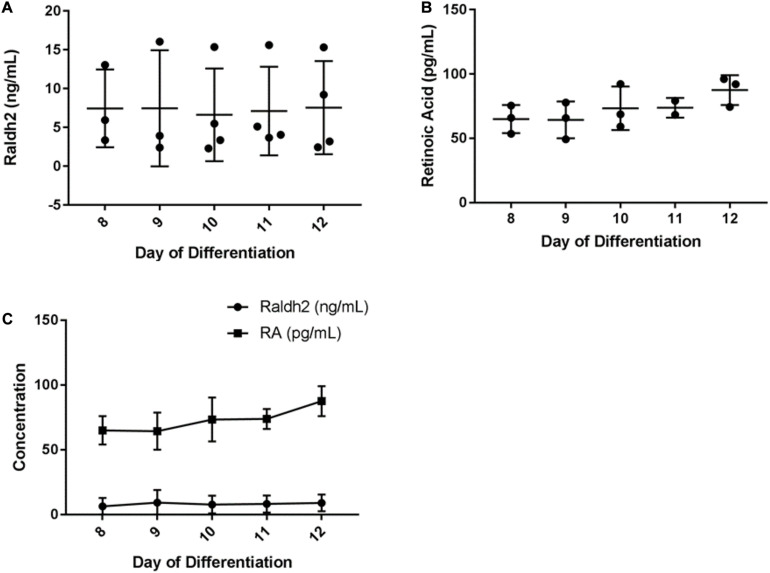
ELISA determination of HGF11-derived MN expression of **(A)** Raldh2 **(B)** RA, and **(C)** comparison of the 2 over 8–12 day period. Error bars: mean and SD. *n* = 4 independent experiments, 3 technical replicates per experiment.

To assess the concentration of Raldh2 and RA produced by HGF11-derived MNs, culture medium from 8–12 day monolayer cultures was tested by competitive ELISA. Results showed that Raldh2 was produced throughout the 8–12 day period, with average concentrations of: D8, 7.45 ng/mL; D9, 7.47 g/mL, D10, 6.63 ng/mL; D11, 7.12 ng/mL, and D12, 7.55 ng/mL (Figure 8A, see also Supplementary Table 4). A one-way ANOVA did not detect a statistically significant difference in the concentration of Raldh2 across the five timepoints [*F*(4,12) = 0.19, *p* = 0.939, df = 4]. Results for RA production also showed production throughout this period, with average concentrations of: D8, 64.98 ng/mL (216 pM); D9, 64.32 ng/mL (214 pM); D10, 73.36 ng/mL (244 pM); D11, 73.76 ng/mL (246 pM) and D12: 87.53 ng/mL (291 pM) (Figure 8B and Supplementary Table 5). One-way ANOVA did not detect a statistically significant difference in the concentration of RA in the culture medium across the five timepoints [*F*(4,9) = 1.53, *p* = 0.274, df = 4]. There was no correlation between the concentration of Raldh2 and RA (*p* = 0.337) (Figure 8C). The concentration of RA produced by the HGF11 derived MNs was much lower than the *in vivo* concentration of 122 nM reported for the E9.5 neural tube (Horton and Maden, 1995). Although the concentration was low, it remained consistent over the five day period tested. Therefore, the HGF11 derived MNs can potentially be used as a stable source of RA to drive neural differentiation *in vitro*, which addresses the limitation of RA light-degradation. Most neural differentiation protocols for mESC last for 5 days and supplement RA on days 2 and 4 of differentiation. HGF11 derived MNs produced RA over a five day period and could be a sufficient neural inductor for the CGR8.

### Non-localized Cell-Derived Endogenous RA Does Not Induce Differentiation

**FIGURE 9:**
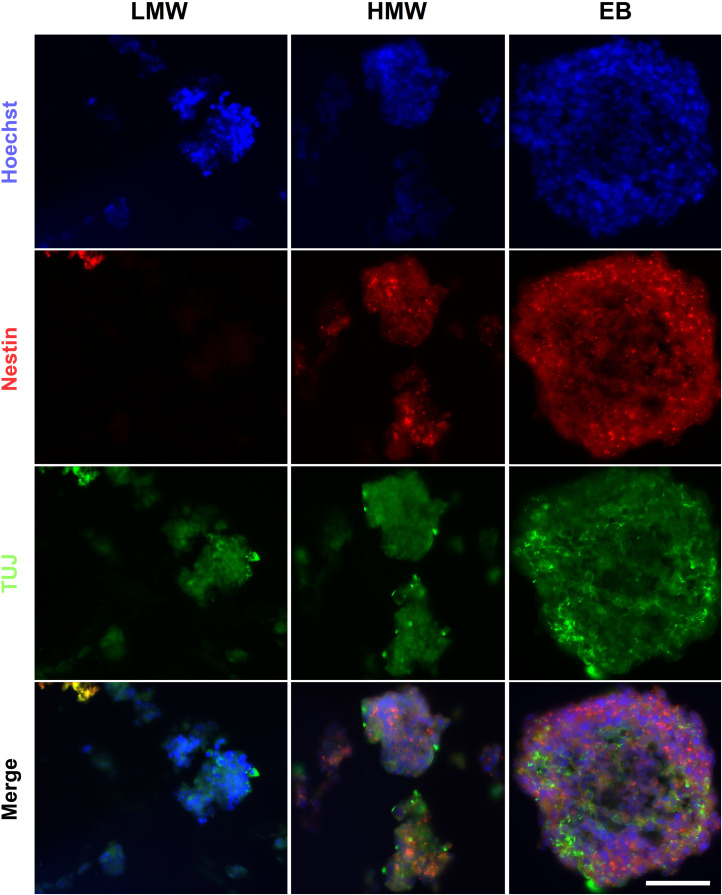
CGR8 differentiation in Boyden chamber co-cultures with RA producing HGF11 MN. Representative ICC images of markers of the 3 germ layers in EBs and aggregates of CGR8 cells encapsulated in LMW and HMW alginate beads. Images collected from *n* = 4 independent experiments. Scale Bar: 50 μm.

Okada et al. (2004) reported that RA concentrations from 1 nM to 1 μM were sufficient to induce ESC differentiation toward the three germ layers, however, 1 nM concentrations induced more mesodermal differentiation, and higher concentrations induced more neural differentiation. Furthermore, Ulven et al. (2001) quantified RA in different spinal cord regions of E12.5 mouse embryos: brachial (74 nM), thoracic (36 nM), and lumbar (200 nM). These RA concentrations are all substantially higher compared to the pM concentrations we observed in the medium, using ELISA. However, sequestered RA (intracellular RA bound to Retinoic Acid Receptors) concentration is higher inside cells. Therefore, it is possible that RA from HGF11-derived LMC-MNs can drive ESCs differentiation in alginate beads, if the RA source is placed in close proximity to the target ESCs. Nevertheless, in order to examine potential effects of non-localized RA delivery, we performed a co-culture experiment with HGF11-derived MN monolayers and CGR8 ESCs in alginate beads in Boyden chambers (Supplementary Figure 5A). LMW and HMW alginate beads contained very few aggregates by day 6 and those observed were primarily near the bead edge (Supplementary Figure 5B). Immunocytochemistry revealed limited Nestin and βIII-tubulin expression in alginate beads in contrast to widespread expression in EB controls generated using 1 μM exogenous RA (Figure 9). This suggests that the concentration of LMC-MN-derived RA present within the culture medium is not sufficient to induce ESC aggregation and neural differentiation in this experimental setup. This result was expected as the RA synthesized by LMCm-MNs *in vivo* acts in a localized manner on nearby LMCl-MNs, and does not diffuse over large distances during development.

### Fiber Alginate Scaffold Has Potential to Investigate Patterned Differentiation

Neural induction of ESC under RA gradients has been investigated *in vitro via* hydrogel microfluidic systems. Cosson and Lutolf (2014) highlight the correlation between increased RA concentration and normalized GFP intensity of the Sox1-GFP knock-in reporter ESC line. To investigate whether a localized concentration gradient of RA derived from LMC-MNs could induce patterned differentiation, CGR8 ESCs and HGF11-derived LMC-MNs were co-encapsulated within alginate fibers. LMW alginate was chosen due to its lower viscosity, allowing the two cell suspensions in the gradient maker chambers to be mixed during alginate fiber generation (Figure 1A). For this experiment, both chambers had cell suspensions containing equal cell densities of CGR8 ESCs resulting in an alginate fiber with a constant cell density of CGR8 ESCs and a decreasing cell density of HGF11 ESC-derived MNs along the length. Therefore, the resulting alginate fibers contained a graded cell density of HGF11-derived LMC-MNs along the length of the fiber, providing a localized concentration gradient of RA to the constant (non-gradient) cell density of encapsulated CGR8 ESCs.

**FIGURE 10:**
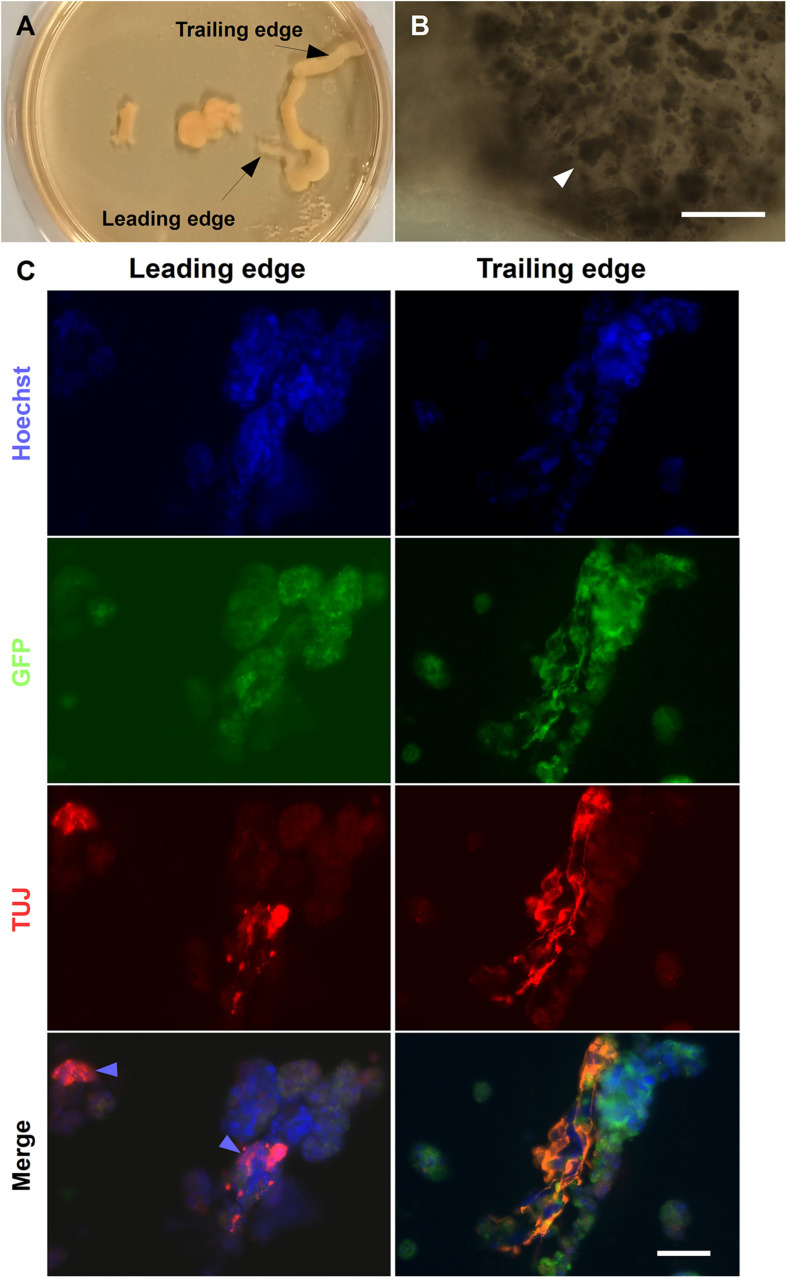
Neural differentiation in alginate tubular scaffold co-cultures. **(A)** Brightfield images of alginate fibers formed using a gradient maker. **(B)** High magnification images of the leading sections of a fiber on day 6 showing encapsulated aggregates (white arrowheads). Scale Bar: 400 μm. **(C)** Alginate fibers contained ectoderm marker βIII-tubulin. The fibers contained TUJ1^+^ cells at both the ends by day 6 of differentiation. However, TUJ^+^/GFP^−^ cells can be seen predominantly at the leading end of the fiber, where most MNs are localized (blue arrowhead). Scale Bars: Top right panel: 400 μm, bottom right panel: 50 μm.

**FIGURE 11:**
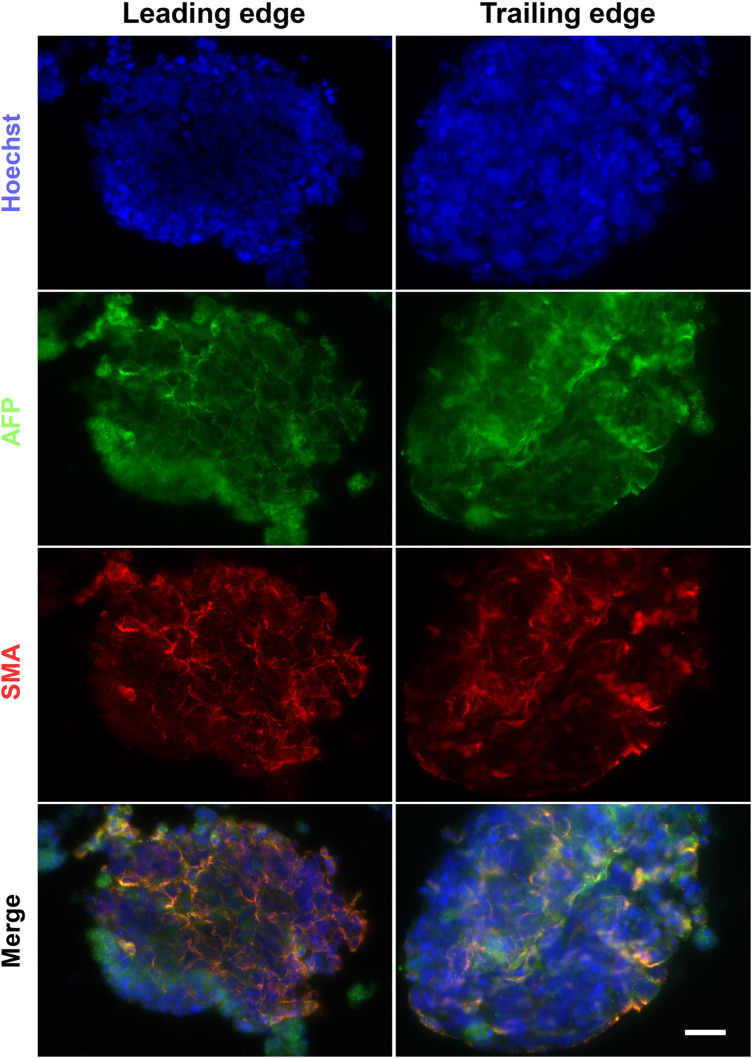
Endoderm and mesoderm differentiation in alginate fibers. The alginate fibers contained markers of endoderm and mesoderm germ layers at day 6 of differentiation. These were present at both the trailing and leading end of the fiber. Scale Bar: 50 μm.

The alginate fibers had a variable diameter along their length, and swelled over the course of the culture, but predominantly remained intact by day 6 (Figure 10A). Brightfield images of the leading and trailing ends of the fibers showed that the encapsulated cells had formed aggregates (Figure 10B), with some free-floating aggregates in the culture medium (data not shown). The presence of aggregates inside the fibers suggests that, unlike the encapsulated ESCs in the Boyden chamber co-culture experiments, ESCs encapsulated in alginate fibers may be exposed to sufficient levels of RA from HGF11-derived LMC-MNs to aggregate. However, our current experimental paradigm cannot exclude effects through direct cell-cell (CGR8:LMC-MN) interactions (Rhee et al., 2019) or the possibility of undifferentiated HGF11 aggregates. We did determine whether aggregates underwent differentiation and potential effects of the graded concentrations of LMC-MNs and the RA derived thereof, by cryosectioning and immunostaining the trailing and leading ends of the fibers for markers of the three germ layers. Markers of all three germ layers (AFP, SMA, and βIII-tubulin) were observed at both high and low LMC-MN levels (leading and trailing end, respectively) of the alginate fibers (Figures 10C, 11). However, only in the leading end of the fibers did we observe cells that were simultaneously TuJ1^+^ and GFP^–^ (Figure 10C, blue arrowheads). These cells correspond to CGR8-derived neurons, which differentiated probably under the influence of RA produced by the HGF11-derived LMC-MNs. This indicates that the RA produced was sufficient to induce cell differentiation of the encapsulated aggregates. From immunofluorescence images, we did not detect any evident difference in differentiation at the trailing versus leading edge of the fibers at a qualitative level. Cells from all three germ layers were present, with mesoderm (SMA) and endoderm (AFP) being predominant (Figure 11). However, future quantification will be required to determine precise compositions.

## Conclusion

In this study, we investigated the aggregation and differentiation of mouse ESCs encapsulated in alginate scaffolds of different molecular weights. A driving hypothesis behind our experiments was that a direct comparison of alginate hydrogels of different G-monomer content would identify optimal formats for neural differentiation. In contrast, the results revealed no significant difference between HMW and LMW alginates in mouse ESC cell aggregation, viability or differentiation. Our results illustrate that aggregates in alginate beads are similar to canonical EBs and contain cells from all three germ layers. Mouse ESCs will differentiate in both high G- and high M-residue alginates, which enables examination of cell-cell and cell-matrix interactions, within the context of aggregation and differentiation. Future studies will build on our data to fine-tune the platform for specific applications and desired cell populations. Potential parameters that can be modulated include: alginate density and functionalization, as well as organizer cell line that drives the differentiation of encapsulated ESCs.

The universal application of morphogens in a differentiation medium to induct ESCs oversimplifies developmental processes and limits experimental outcomes to binary outputs. We used the transgenic ESC line HGF11 to derive LMCm-MNs, as a potential *in situ* source of RA. Expression levels of Raldh2 and RA by LMCm-MNs from the transgenic ESC line HGF11 were consistent throughout a 5 day period but below physiological levels. Therefore, they could not drive ESC aggregate formation in a Boyden chamber co-culture paradigm. However, co-culture by graded co-encapsulation in alginate fibers did permit aggregate formation and subsequent stem cell differentiation. Further work will be required to confirm whether EB-like aggregate formation and subsequent neuronal differentiation are driven by local RA from LMCm-MNs or other factors, such as cell-cell interactions. Our current results serve as proof-of-principle for future development of the alginate tubular platform to enable detailed investigations of patterned differentiation.

## Data Availability Statement

The datasets generated for this study are available on request to the corresponding author.

## Author Contributions

OF: conceptualization, formal analysis, investigation, and writing—review and editing. AB: resources, supervision, validation, and writing—review and editing. BW: resources and supervision. ED: conceptualization, data curation, funding acquisition, final approval of the manuscript, project administration, resources, supervision, writing—original draft, and writing—review and editing. All authors contributed to the article and approved the submitted version.

## Conflict of Interest

The authors declare that the research was conducted in the absence of any commercial or financial relationships that could be construed as a potential conflict of interest.

## Supplementary Material

Supplementary Figure 1 Diameter (in μm) of HMW and LMW alginate beads over an incubation period of 6 days. Differences between day groups (*N* = 3 for each day) were statistically significant, as determined by a one-way ANOVA [HMW: *F*(3,8) = 10.9476, *p* = 0.0033 and LMW: *F*(3,8) = 8.9472, *p* = 0.0062].

Supplementary Figure 2 Spatial variation in β-III-tubulin and Nestin (ectoderm markers) in sequential sections of EBs and HMW/LMW beads. Scale Bars: 50 μm.

Supplementary Figure 3 Spatial variation in AFP and SMA (endoderm and mesoderm markers) in sequential sections of EBs and HMW/LMW beads. Scale Bars: 50 μm.

Supplementary Figure 4 Negative control for the HGF11 differentiation into LMC-MN. CGR8-derived MNs (depicted) were HB9^+^ and GFP^–^, demonstrating that the GFP expression under the control of HB9 was specific to the transgenic HGF11 cell line. The control condition contained GFP^–^, HB9^+^, and Foxp1^+^ cells (arrowheads) which was expected, due to spontaneous differentiation to an LMC lineage. Scale Bar: 20 μm.

Supplementary Figure 5 Co-culture experiment with HGF11-derived MN monolayers. **(A)** Diagram of the Boyden chamber containing the alginate encapsulated mESC and the HGF11-derived MN. **(B)** Brightfield images of alginate beads with encapsulated CGR8, after 6 days of co-culture with HGF11-derived MNs. The encapsulated CGR8 cells generated very few aggregates by day 6 in both HMW and LMW beads. Scale bars: 400 μm.

Supplementary Table 1 Details of antibodies used for immunocytochemistry experiments.

Supplementary Table 2 Product details for LMW and HMW alginate solutions.

Supplementary Table 3 Details of primers used for quantitative PCR.

Supplementary Table 4 HGF11-derived MNs produce Raldh2.

Supplementary Table 5 HGF11-derived MNs produce RA.
