# Supplementary figures and images for: A Fiber Alginate Co-culture Platform for the Differentiation of mESC and Modeling of the Neural Tube
Source: Front Neurosci. 2021 Jan 12;14:524346. doi: 10.3389/fnins.2020.524346 (PMC7835723; doi:10.3389/fnins.2020.524346)

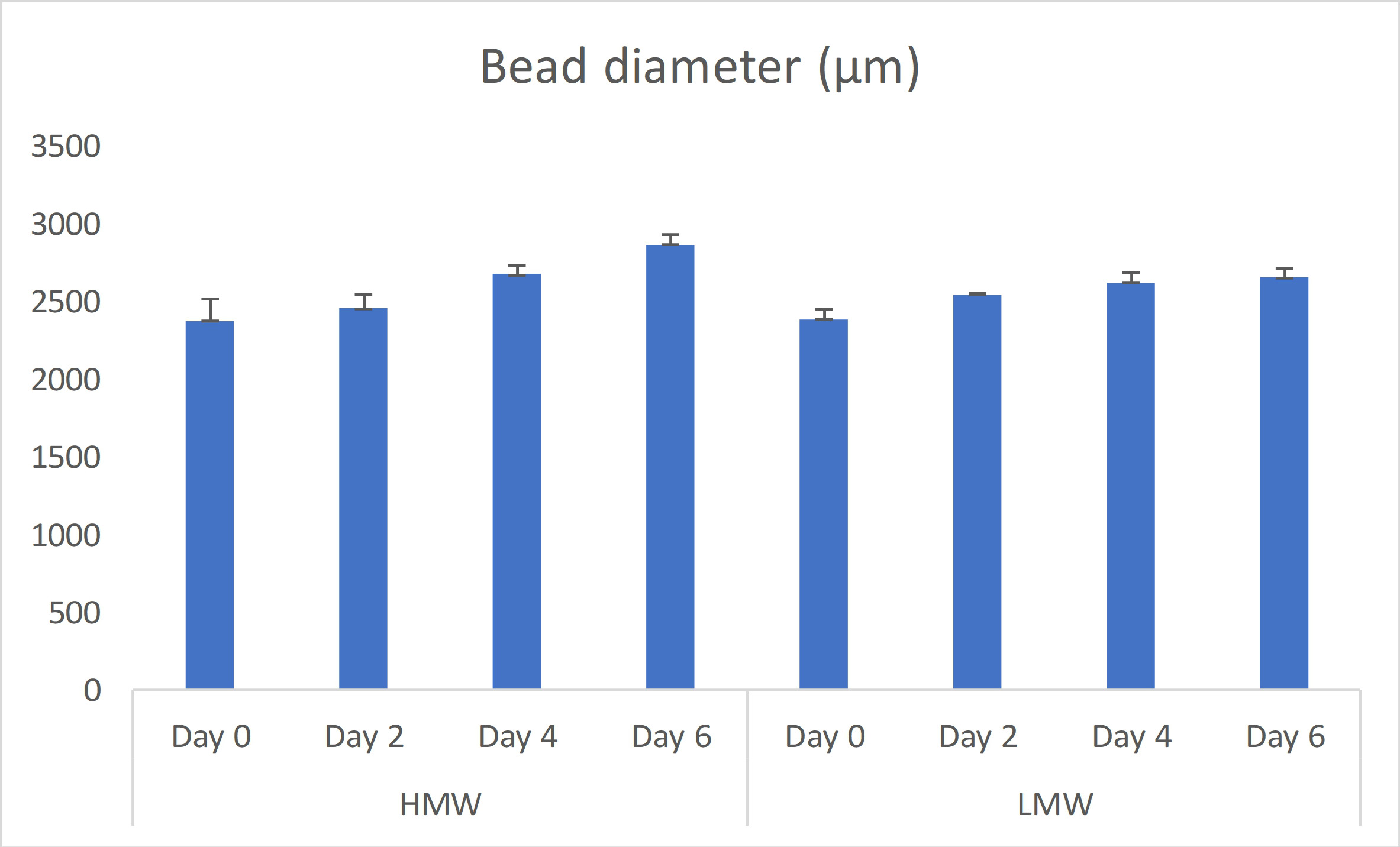

Supplement: Supplementary Figure 1 — Diameter (in μm) of HMW and LMW alginate beads over an incubation period of 6 days. Differences between day groups (N = 3 for each day) were statistically significant, as determined by a one-way ANOVA [HMW: F(3,8) = 10.9476, p = 0.0033 and LMW: F(3,8) = 8.9472, p = 0.0062]. [file Image_1.JPEG]

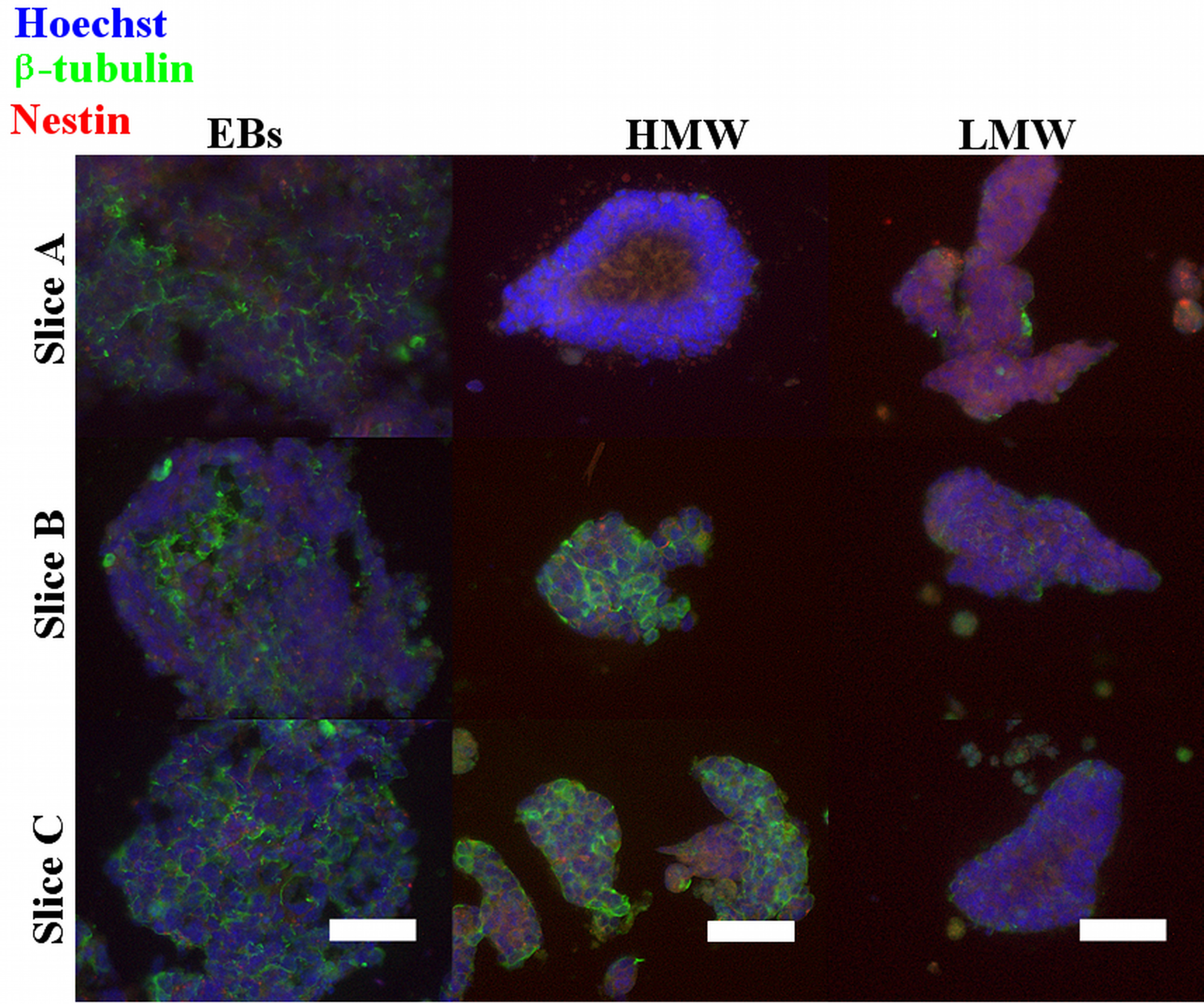

Supplement: Supplementary Figure 2 — Spatial variation in β-III-tubulin and Nestin (ectoderm markers) in sequential sections of EBs and HMW/LMW beads. Scale Bars: 50 μm. [file Image_2.JPEG]

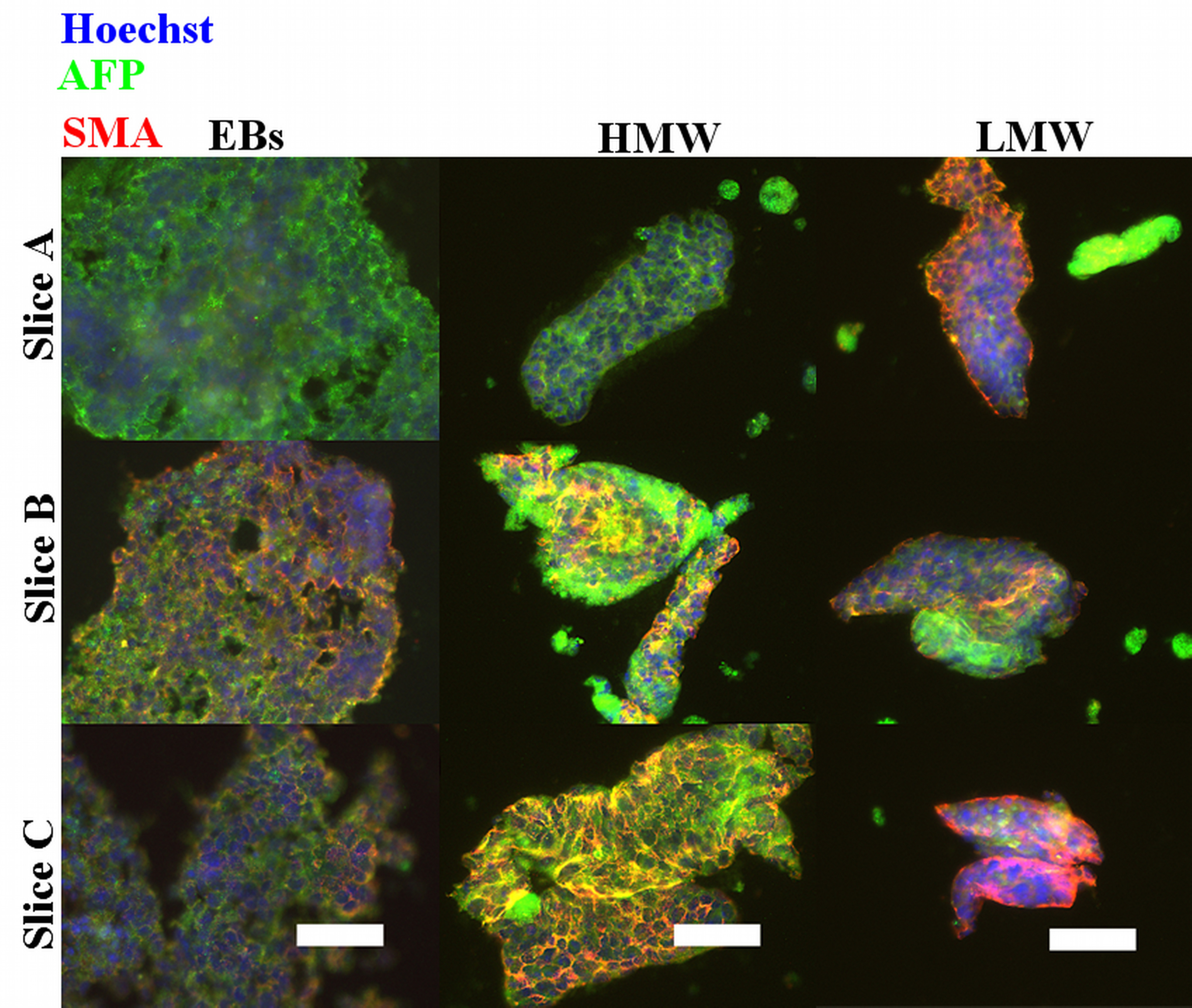

Supplement: Supplementary Figure 3 — Spatial variation in AFP and SMA (endoderm and mesoderm markers) in sequential sections of EBs and HMW/LMW beads. Scale Bars: 50 μm. [file Image_3.JPEG]

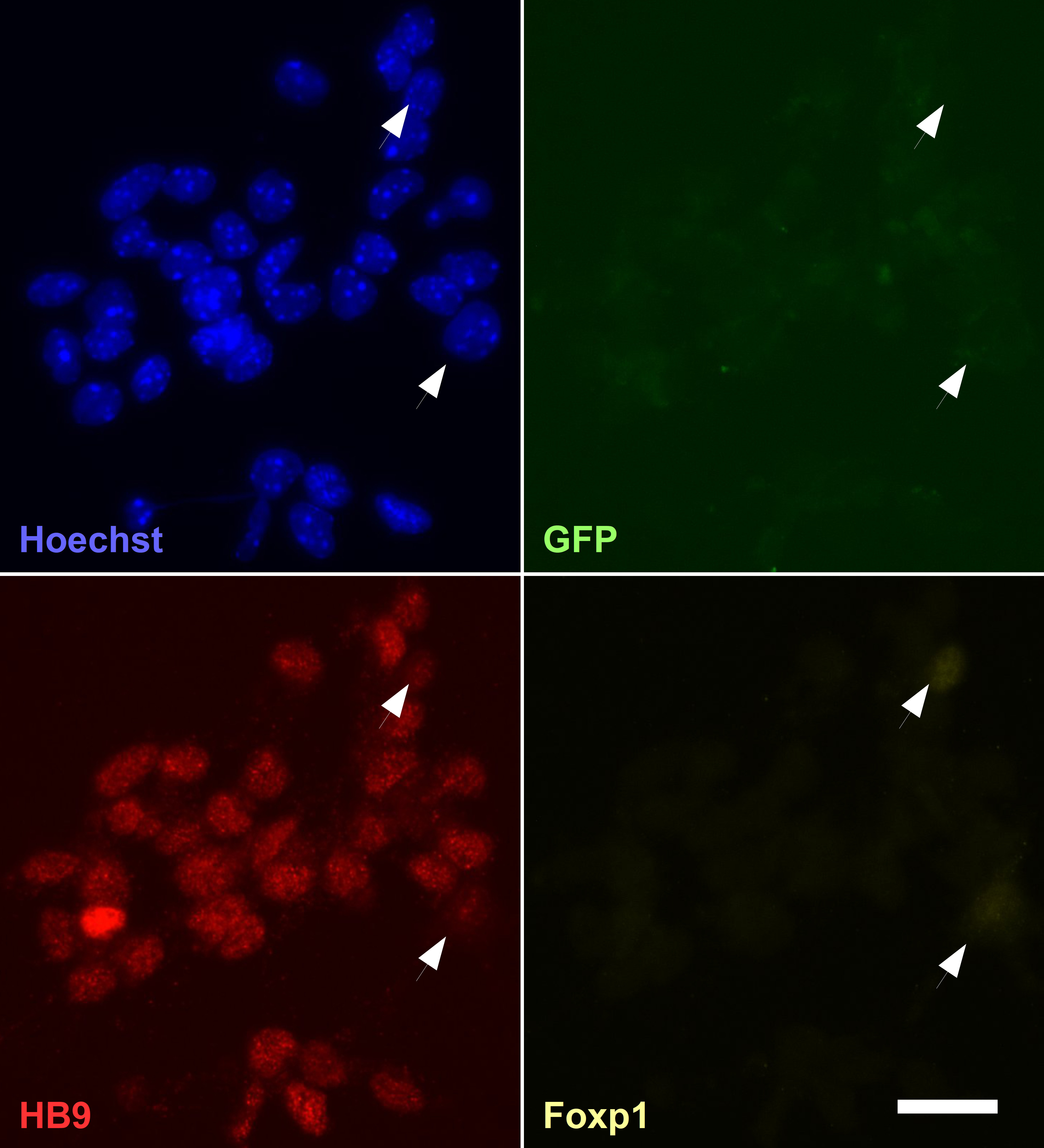

Supplement: Supplementary Figure 4 — Negative control for the HGF11 differentiation into LMC-MN. CGR8-derived MNs (depicted) were HB9+ and GFP–, demonstrating that the GFP expression under the control of HB9 was specific to the transgenic HGF11 cell line. The control condition contained GFP–, HB9+, and Foxp1+ cells (arrowheads) which was expected, due to spontaneous differentiation to an LMC lineage. Scale Bar: 20 μm. [file Image_4.JPEG]

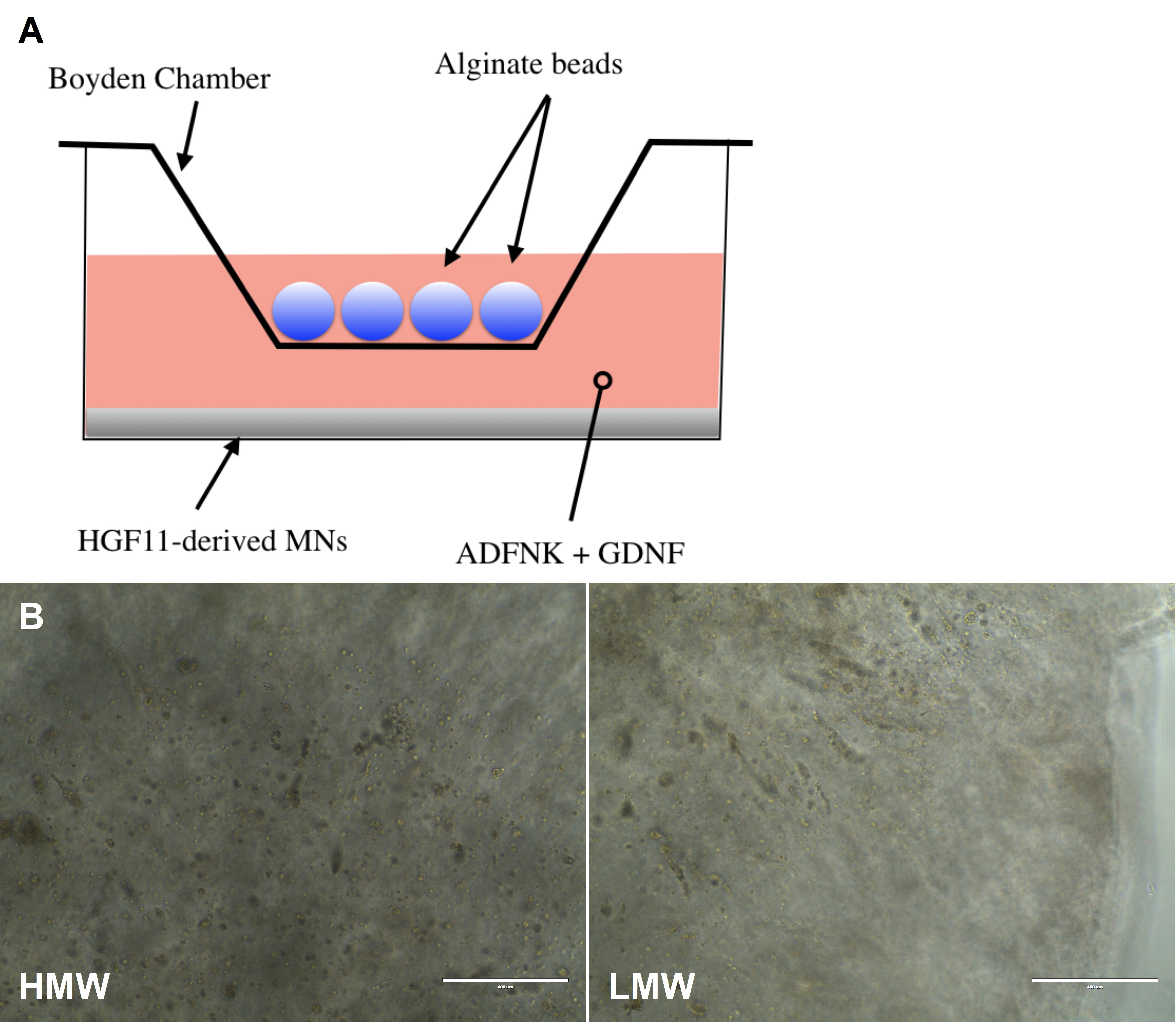

Supplement: Supplementary Figure 5 — Co-culture experiment with HGF11-derived MN monolayers. (A) Diagram of the Boyden chamber containing the alginate encapsulated mESC and the HGF11-derived MN. (B) Brightfield images of alginate beads with encapsulated CGR8, after 6 days of co-culture with HGF11-derived MNs. The encapsulated CGR8 cells generated very few aggregates by day 6 in both HMW and LMW beads. Scale bars: 400 μm. [file Image_5.JPEG]
