# Supplementary material for: A Fiber Alginate Co-culture Platform for the Differentiation of mESC and Modeling of the Neural Tube
Source: Front Neurosci. 2021 Jan 12;14:524346. doi: 10.3389/fnins.2020.524346 (PMC7835723; doi:10.3389/fnins.2020.524346)
Supplement: Supplementary Table 1 — Details of antibodies used for immunocytochemistry experiments. [file Table_1.docx]

Table ST1: Details of antibodies used for immunocytochemistry experiments

| **Primary Antibody** | **Supplier** | **Code** | **Host** | **Concentration** |
| --- | --- | --- | --- | --- |
| a-fetoprotein | Thermo Fisher | PA5-21004 | Rabbit IgG | 1:200 |
| α-smooth muscle actin | Abcam | ab7817 | Mouse IgG2a | 1:300 |
| α-smooth muscle actin | Abcam | ab5694 | Rabbit IgG | 1:300 |
| β-III-tubulin | Abcam | ab41489 | Chicken IgY | 1:300 |
| β-III-tubulin | Abcam | ab18207 | Rabbit IgG | 1:300 |
| Foxp1 | Thermo Fisher | PA5-52006 | Rabbit IgG | 1:100 |
| HB9 | Santa Cruz | sc515769 | Mouse IgG2a | 1:200 |
| HB9 | Thermo Fisher | PA5-23407 | Rabbit IgG | 1:150 |
| Nestin | Santa Cruz | sc101541 | Rat IgG | 1:250 |
| **Secondary Antibody** | **Supplier** | **Code** | **Host** | **Concentration** |
| anti-chicken Alexa Fluor 647 | Abcam | ab150171 | Goat IgY | 1:500 |
| anti-chicken IgY fluorescein | Aves Labs | F1005 | Goat IgY | 1:500 |
| anti-mouse Alexa Fluor 488 | Life Tech | A11001 | Goat IgG | 1:500 |
| anti-mouse Alexa Fluor 568 | Life Tech | A11004 | Goat IgG | 1:500 |
| anti-rabbit Alexa Fluor 488 | Life Tech | A27034 | Goat IgG | 1:500 |
| anti-rabbit Alexa Fluor 568 | Life Tech | A11011 | Goat IgG | 1:500 |
| anti-rabbit Alexa Fluor 647 | Life Tech | A21245 | Goat IgG | 1:500 |
| anti-rat Alexa Fluor 594 | Life Tech | A11007 | Goat IgG | 1:500 |
| Hoechst 33342 10mg/ml | Life Tech | H3570 | n/a | 1:20,000 |
