# Supplementary material for: A Fiber Alginate Co-culture Platform for the Differentiation of mESC and Modeling of the Neural Tube
Source: Front Neurosci. 2021 Jan 12;14:524346. doi: 10.3389/fnins.2020.524346 (PMC7835723; doi:10.3389/fnins.2020.524346)
Supplement: Supplementary Table 2 — Product details for LMW and HMW alginate solutions. [file Table_2.docx]

Table ST2: Product details for LMW and HMW alginate solutions

| Product | LMW | HMW |
| --- | --- | --- |
| Supplier | Sigma #71238 | Acros Organics #17777 |
| Viscosity (at 1% w/v) | 100-200 mPas | 350-550 mPas |
| Molecular weight | 100,000-200,000g/mol | 450,000-550,000g/mol |
| G:M content | 65-75%:35-25% | 40%:60% |
