# Supplementary material for: A Fiber Alginate Co-culture Platform for the Differentiation of mESC and Modeling of the Neural Tube
Source: Front Neurosci. 2021 Jan 12;14:524346. doi: 10.3389/fnins.2020.524346 (PMC7835723; doi:10.3389/fnins.2020.524346)
Supplement: Supplementary Table 3 — Details of primers used for quantitative PCR. [file Table_3.docx]

Table ST3: Details of primers used for quantitative PCR

| **Name** | **Sequence (5’-3’)** | **bp** | **Tm** | **GC(%)** |
| --- | --- | --- | --- | --- |
| Fwd Pou5f1 | GAAGCCGACAACAATGAGAA | 20 | 62.8 | 45 |
| Rev Pou5f1 | TCTCCAGACTCCACCTCACA | 20 | 63.7 | 55 |
| Fwd Pax6 | CGGAGGGAGTAAGCCAAGAG | 20 | 65.0 | 60 |
| Rev Pax6 | TCTGTCTCGGATTTCCCAAG | 20 | 64.1 | 50 |
| Fwd Nestin | TTGCAGACACCTGGAAGAAG | 20 | 63.1 | 50 |
| Rev Nestin | TCAAGGGTATTAGGCAAGGG | 20 | 62.7 | 50 |
| Fwd b-actin | AGAGGGAAATCGTGCGTGAC | 20 | 66.5 | 55 |
| Rev b-actin | CAATAGTGATGACCTGGCCGT | 21 | 66.0 | 52 |
