# Supplementary material for: A Fiber Alginate Co-culture Platform for the Differentiation of mESC and Modeling of the Neural Tube
Source: Front Neurosci. 2021 Jan 12;14:524346. doi: 10.3389/fnins.2020.524346 (PMC7835723; doi:10.3389/fnins.2020.524346)
Supplement: Supplementary Table 4 — HGF11-derived MNs produce Raldh2. [file Table_4.docx]

Table ST4: HGF11-derived MNs produce Raldh2. Text in red indicates outliers that were excluded from statistical analysis. n=4 experiments, 3 technical replicates per experiment.

|  | **Concentration (ng/mL)** | | | | **Mean** |
| --- | --- | --- | --- | --- | --- |
| D8 | 0.00 | 5.95 | 13.05 | 3.37 | 7.45 |
| D9 | 2.41 | 10.73 | 16.06 | 3.94 | 7.47 |
| D10 | 2.3 | 5.5 | 15.36 | 3.37 | 6.63 |
| D11 | 3.67 | 5.12 | 15.62 | 4.06 | 7.12 |
| D12 | 2.45 | 9.22 | 15.32 | 3.2 | 7.55 |
| R | 0.86 | 0.86 | 0.88 | 1 |  |
