# Supplementary material for: A Fiber Alginate Co-culture Platform for the Differentiation of mESC and Modeling of the Neural Tube
Source: Front Neurosci. 2021 Jan 12;14:524346. doi: 10.3389/fnins.2020.524346 (PMC7835723; doi:10.3389/fnins.2020.524346)
Supplement: Supplementary Table 5 — HGF11-derived MNs produce RA. [file Table_5.docx]

Table ST5: HGF11-derived MNs produce RA. Text in red indicates outliers which were excluded from statistical analysis. n=3 experiments, 3 technical replicates per experiment.

|  | Concentration (ng/mL) | | | Mean | pM |
| --- | --- | --- | --- | --- | --- |
| D8 | 53.59 | 65.9 | 75.47 | 64.98 | 216 |
| D9 | 49.31 | 65.85 | 77.81 | 64.32 | 214 |
| D10 | 59.24 | 68.69 | 92.17 | 73.36 | 244 |
| D11 | 68.32 | 168.88 | 79.21 | 73.76 | 246 |
| D12 | 96.09 | 92.10 | 74.41 | 87.53 | 291 |
| r^2^ | 0.93 | 0.9 | 0.98 |  |  |
